# Epidemiology of Syphilis, Diagnosis, Differential Diagnosis and Treatment: Up-to-Date Knowledge

**DOI:** 10.3390/jcm15197440

**Published:** 2026-09-24

**Authors:** Mateusz Matwiejuk, Agnieszka Beata Serwin, Hanna Mysliwiec, Iwona Flisiak

**Affiliations:** Department of Dermatology and Venerology, Medical University of Bialystok, 15-540 Bialystok, Poland

**Keywords:** syphilis, epidemiology, prevalence, diagnosis, differential diagnosis, treatment

## Abstract

Syphilis is an infectious sexually transmitted disease that has re-emerged as a major global public health concern, with a steadily increasing incidence in many regions of the world. It is primarily spread through sexual contact, but can also be transmitted from mother to foetus during pregnancy and, rarely, through blood transfusion. It is estimated that millions of people are affected by syphilis globally, although the exact number of infectious individuals varies by region and is difficult to determine precisely. Despite advances in diagnostic methods and the availability of effective antibiotic treatment, syphilis continues to pose significant diagnostic and epidemiological challenges. Syphilis has been called “the great imitator” because its clinical manifestations can resemble many common dermatologic conditions, such as psoriasis, lichen planus, eczema, pityriasis rosea, and others. In addition to mimicking skin diseases, syphilis can also present with ocular involvement (ocular syphilis) and hepatic manifestations (syphilitic hepatitis), further complicating diagnosis. This review summarises current knowledge on the epidemiology, diagnosis, differential diagnosis, and treatment.

## 1. Introduction

### 1.1. History and Epidemiology

Syphilis is one of the sexually transmitted infections (STIs), which is caused by *Treponema pallidum subspecies pallidum* (*T. pallidum*) (spirochete bacterium) [1]. Two main hypotheses have been proposed regarding the origin of syphilis. The first one described that it was an endemic disease in the Caribbean region, and it was carried from this location to Europe during the Columbus expedition in the late 1400s. The second one points out that it originated in central Africa and moved to Europe before Christopher Columbus’s voyage [2]. According to the World Health Organisation, approximately 0.6% of the global population had syphilis in 2022. In addition, approximately 8 million new cases were registered in adults between 15 and 49 years of age and around 700,000 cases of congenital syphilis (CS) [3]. In 2024, 41,051 cases of syphilis were reported in 29 European Union and European Economic Area (EU/EEA) countries. The overall rate was 10.8 per 100,000 European population. The highest rate was observed among men aged 25–34 years, with 43 cases per 100,000 population. The incidence was approximately seven-fold higher in men than in women [4]. According to the CDC, reported syphilis cases in the United States increased by 80% between 2018 and 2022, rising from approximately 115,000 to more than 207,000 cases [5].

### 1.2. Bacteriology

*T. pallidum* is a Gram-negative, spiral-shaped, motile bacterium. It measures from 5 to 15 µm in length and approximately 0.20 µm in diameter. It belongs to the family Spirochaetaceae, order Spirochaetales, phylum Spirochaetes [6].

### 1.3. Transmission

Syphilis is transmitted through direct contact with infectious lesions [7], transplacentally from mother to foetus [8], and less commonly through exposure to infected blood or shared needles [9].

Based on clinical manifestations and laboratory findings, the course of syphilis may be divided into latent, primary, secondary, and tertiary stages [10]. Considering the time since primary infection, early syphilis is defined as syphilis occurring within the first year after infection; late syphilis is referred to as syphilis diagnosed more than a year after infection [11].

### 1.4. Clinical Picture

The first stage of syphilis is chancre, located in the genitals or mucocutaneous areas [12]. The average incubation period for the appearance of the chancre is 3 to 90 days, with a median of 21 days [13].

After several weeks to a couple of months following the appearance of the chancre, approximately 25% of patients with untreated infection develop secondary syphilis [14]. Secondary syphilis, also known as ‘the great imitator of skin diseases’, consists of a usually non-pruritic rash—which may affect the palms and soles—and is accompanied by fever and lymphadenopathy [15]. The skin symptoms comprise a widespread, symmetric, macular or papular eruption located over the entire trunk and extremities [16]. In addition to these skin lesions, patients with secondary syphilis may experience ‘moth-eaten’ alopecia—typically situated on the scalp, eyebrows, or beard [17]. Vitiligo, in the course of secondary syphilis, presents as hypopigmented patches, most commonly located on the scalp, face, neck, forearms, dorsal hands, fingers, and feet [18]. Moreover, in the course of secondary syphilis, condyloma lata appear, which are hypertrophic papules or plaques with a flat surface. They occur more commonly in flexural areas, especially in the anogenital region [19].

The next phase of syphilis, after secondary syphilis, is late syphilis, which appears in approximately 25% to 40% of patients with untreated infection. The clinical manifestations may occur at any time from 1 to 30 years after the primary infection [20].

Late syphilis may be divided into asymptomatic latent late syphilis and symptomatic tertiary syphilis, with clinical manifestations depending on the organ affected, such as cardiovascular syphilis [21]. It has been estimated that nearly 25% of patients with tertiary syphilis may develop cardiovascular disorders [22]. Cardiovascular syphilis primarily involves inflammation and enlargement of the aorta, occurring decades after the initial infection [23]. However, the most common disorders in cardiovascular syphilis include syphilitic aortic aneurysm, syphilitic aortic regurgitation, syphilitic aortitis, syphilitic coronary ostial stenosis, and syphilitic gummas of the myocardium [24].

Another manifestation of tertiary syphilis is gummatous syphilis. Gummas are granulomatous, nodular lesions ranging from millimetres to centimetres in length. These are very uncommon pathological abnormalities. Gummas can arise in various organs—most often in bone, liver, mucocutaneous sites, skin, and testes [25]. In particular, granulomatous neurosyphilis is a rare clinical presentation, due to widespread administration of penicillin. However, it poses a diagnostic challenge and is very easy to confuse with cerebral toxoplasmosis [26].

Regarding the central nervous system, around 25 years after the primary infection, two severe conditions may occur: general paresis and tabes dorsalis [27].

Regardless of the stage of syphilis, neurological, ophthalmological, and otolaryngological symptoms may appear at any stage of the disease. These symptoms warrant diagnostic evaluation to rule out neurosyphilis [28].

### 1.5. Congenital Syphilis

Congenital syphilis (CS) appears when *Treponema pallidum* is transmitted from a mother with syphilis to her foetus during pregnancy [29]. The consequences of CS are severe. Perinatal mortality reaches nearly 40% of cases, including miscarriage, neonatal death, and stillbirth. Interestingly, approximately 15% of newborns may exhibit syphilitic symptoms [30]. Early CS refers to clinical manifestations that appear in the first two years of life, compared with those occurring after two years of age, which are designated as late CS [31]. The symptoms of early CS may vary from asymptomatic ones (around 70% of cases) to stillbirth (circa 40% in patients with untreated pregnancies) [32]. Additional manifestations of this phase of CS include pneumonia, snuffles, lymphadenopathy, hepatosplenomegaly, cholestasis, hyperbilirubinaemia, maculopapular rash, and desquamation on the palms and soles. Meningitis and seizures may be present during central nervous system involvement [33]. Regarding skeletal system involvement, osteochondritis and periostitis may occur, with or without pseudoparalysis of Parrot, which is a reduced range of movement due to painful periostitis [34]. In the late stage of CS, the clinical presentation includes the triad of CS: interstitial keratitis, sensorineural hearing loss, and notched central incisors (Hutchinson’s teeth) [35]. Moreover, patients in this phase may develop anterior bowing of the shin (sabre shin), painless swelling of the knee (called Clutton’s joints), mulberry molars (which are first molars with rudimentary cusps), and saddle nose. In addition to the above-mentioned symptoms, uveitis or optic atrophy and developmental delays are rarely present [33].

### 1.6. Syphilis Treatment

Benzathine benzylpenicillin G (BPG) administered via intramuscular injection remains the first-choice treatment for all stages of syphilis, except for syphilis involving the central nervous system (CNS). In early syphilis, BPG is administered as a single intramuscular (i.m.) dose of 2.4 million units. In late syphilis, benzathine benzylpenicillin G (BPG) is administered as 2.4 million units i.m. once weekly for three consecutive weeks [11,28,36].

An alternative treatment for early syphilis is procaine benzylpenicillin 0.6 million units intramuscularly (i.m.) once daily for 10–14 days, and that for late syphilis is procaine benzylpenicillin 0.6 million units i.m. once daily for 17–21 days [28,36].

Alternative treatments for syphilis include doxycycline 100 mg orally (p.o.) twice daily (for 14 days in early syphilis and for 28 days in late syphilis) and ceftriaxone 1–2 g intramuscularly (i.m.) or intravenously (i.v.) once daily (for 14 days in early syphilis) [28].

If a patient is allergic to penicillin, desensitisation to the antibiotic followed by the first-line regimen is highly recommended [11,28,36].

In CNS syphilis, the drug of choice is aqueous crystalline penicillin, which is administered intravenously. Benzyl (crystalline) penicillin for CNS syphilis is administered intravenously (i.v.) at a dose of 18–24 million units per day, in divided doses every 4 h, for 10–14 days [28,36,37].

Pregnant women with syphilis should be treated with benzathine benzylpenicillin G (BPG) according to the regimen recommended for their stage of infection [28,36,38].

According to the up-to-date guidelines on CS, the recommended treatment is aqueous crystalline penicillin 100,000–150,000 units/kg body weight/day, which is administered as 50,000 units/kg body weight/dose intravenously every 12 h in the first 7 days of life and for the next 3 days, every 8 h, for a total duration of 10 days [28,36,39].

According to the guidelines for syphilitic patients with HIV infection, treatment should be administered in the same manner as for patients without HIV infection [28,36,40].

However, an interesting study was conducted by Blank et al. [41]. They reported the range of probabilities for serological failure in patients with syphilis and HIV infection treated with 2.4 million units (MU) of intramuscular benzathine penicillin, which ranged from 6.9% (95% CI 2.6% to 14.4%) to 22.4% (95% CI 11.7% to 36.6%). For late latent syphilis treated with 7.2 MU of benzathine penicillin, the failure rates ranged from 19.4% (95% CI 11.9% to 28.9%) to 31.1% (95% CI 22.3% to 40.9%). Failure estimates with 18–24 MU of aqueous penicillin for the treatment of neurosyphilis ranged from 27.3% (95% CI 6.0% to 61.0%) to 27.8% (95% CI 14.2% to 45.2%) [41].

The goal of this paper is to focus on the latest epidemiologic data and diagnostic procedures used to confirm syphilis at different stages.

## 2. Materials and Methods

A medical literature search of PubMed (1990–present), Google Scholar, and Embase was conducted in the autumn and winter of 2025 and 2026, using appropriate terms without date limitations. The main objective of the research was to identify the exact epidemiology of syphilis worldwide and to compile information on diagnosis and differential diagnosis of syphilis. Medical subject headline terms included “syphilis and epidemiology”, “syphilis and prevalence”, “syphilis and diagnosis”, “syphilis and differential diagnosis”, “syphilis and treatment”.

In this article, in the process of looking at the articles searching, we tried to focus mainly on population/participants, intervention/exposure, comparison/control, outcomes, study design, language, publication date, and setting.

Papers with low clinical significance and duplicated publications were excluded from the analysis. Originally, human and animal studies were included in this narrative review. The results of the search strings were combined and removed. Afterwards, the titles and abstracts of the studies searched were independently reviewed by two authors (M.M. and A.B.S.) to identify relevant articles that addressed the review subject. Disagreements between reviewers were resolved by a third reviewer (H.M.). Finally, the selected eligible articles were fully reviewed.

## 3. Discussion

### 3.1. Epidemiology

According to the World Health Organisation (WHO) data, the estimated global prevalence of active syphilis in 2022 was 0.6% (95% CI: 0.5–0.7%) in women and 0.6% (95% CI: 0.5–0.7%) in men [3]. A modelling study published in 2024 estimated the global number of people living with syphilis at approximately 70.5 million in 2021 [42]. The number of cases and age-standardised incidence (ASIR) increased from 160.03 to 178.48 per 100,000 population (from 8,845,220 in 1990 to 14,114,110 in 2019) [43]. In 2022, the WHO Region of the Americas accounted for the highest estimated number of new syphilis cases (3.37 million), representing approximately 42% of all new cases worldwide [44].

The COVID-19 pandemic also influenced reported syphilis epidemiology. According to Welc et al. [45], there was a 14% decrease in syphilis reported cases in various regions worldwide. The observed decrease was attributed to restricted access to healthcare services and the implementation of social distancing measures [45].

Overall, the global burden of syphilis has grown significantly over the past few decades, though COVID-19 pandemic-related disruptions caused a temporary drop in reported cases.

### 3.2. Europe

#### 3.2.1. During the COVID-19 Pandemic

In Europe, the impact of the COVID-19 pandemic on syphilis epidemiology varied considerably between countries. In Denmark, the monthly incidence of syphilis remained comparable between 2019 and 2020 [46]. Similarly, no significant difference in the number of positive syphilis cases was observed in Northern Finland in 2020 (n = 17) compared with the annual numbers reported between 2015 and 2019, whereas a significant decrease was documented in 2021 (n = 7) [47].

In contrast, a significant reduction in newly reported syphilis cases was observed in Greece, where 306 cases were reported in 2020 compared with 340 in 2019, corresponding to a 10% decrease (*p* = 0.02) [48]. In Hungary, Lőrincz et al. also reported changes in syphilis epidemiology during the COVID-19 pandemic; however, the reported numbers appear inconsistent [49].

Italian studies yielded divergent findings. Bonato et al. found no significant differences in the number of cases, patient age, male-to-female ratio, or disease stage between 2016 and 2020 [50]. Conversely, Latini et al. [51] reported that, in Rome, the incidence of syphilis during the first quarter of 2020 approximately doubled among people living with HIV (PLWH) and quadrupled among men who have sex with men (MSM) compared with the corresponding period in 2019. Between 1 January and 9 March 2020, 68 new cases were diagnosed, of which 60 (88%) occurred in MSM and 38 (56%) in PLWH, whereas only 25 cases were diagnosed during the same period in 2019 [51].

In north-eastern Poland, the number of hospitalisations and outpatient visits for sexually transmitted infections declined during the pandemic (March 2020–April 2021) by 83.3% and 41.9%, respectively, compared with the pre-pandemic period (January 2019–February 2020). Despite the reduced number of consultations, the proportion of confirmed syphilis cases per visit was higher during the pandemic than before it (39.4% vs. 28%) [52].

An increase in reported cases was observed in Belgrade, Serbia, where 125 new cases were diagnosed in 2020 compared with 89 in 2019 and 100 in 2018 [53].

In Spain, findings differed by region. In Valencia, the incidence rate decreased to 2.29 cases per 100,000 inhabitants (95% CI: 0.24–7.22) in 2020 compared with 2019–8.32 cases per 100,000 inhabitants [54], whereas in Catalonia the number of reported cases declined from 2207 in 2019 to 1770 in 2020, followed by a partial increase to 1951 cases in 2021 [55].

In contrast, the Jönköping Region in Sweden recorded 25 newly diagnosed cases during the pandemic period (April 2020–September 2021), compared with 10 cases in the pre-pandemic period (October 2018–March 2020) [56].

Overall, European studies demonstrated heterogeneous trends in syphilis incidence during the COVID-19 pandemic, with some countries reporting a temporary decline in notified cases, whereas others observed stable or even increasing numbers, particularly in high-risk populations such as MSM and PLWH.

#### 3.2.2. After the COVID-19 Pandemic

Between 2015 and 2024, notification rates more than doubled [4]. According to the ECDC, notification rates more than doubled between 2015 and 2024. Across 29 EU/EEA countries, the number of reported syphilis cases increased from 26,643 in 2019 to 45,577 in 2024, despite a temporary decline observed in 2020 during the COVID-19 pandemic [4].

Barbaric et al. [57] reported that, between 2015 and 2020, the total number of reported syphilis cases per 100,000 population ranged from 0.4 to 26.5 in 18 non-EU/EEA countries in the Central and Eastern region, as defined by the WHO and the European Centre for Disease Prevention and Control (ECDC). The analysed countries included Albania, Armenia, Azerbaijan, Belarus, Bosnia and Herzegovina, Georgia, Kazakhstan, North Macedonia, Kyrgyzstan, the Republic of Moldova, Montenegro, Serbia, Tajikistan, Turkey, Turkmenistan, the Russian Federation, Ukraine, and Uzbekistan [57].

In France, Laperche et al. [58] reported that between 2015 and 2022, the rate of syphilis seropositivity among blood donors (BDs) increased substantially in 2022, predominantly among males, particularly MSM. Until 2021, syphilis positivity rates remained stable, with a mean of 1.18 per 10,000 donations (range: 1.01–1.38). However, a significant increase was observed in 2022, with a positivity rate of 1.74 per 10,000 donations (*p* = 0.02). Over the entire study period, the syphilis seropositivity rate was 2.2 times higher in male BDs than in female BDs, and this disparity was even greater in 2022, with rates 4.1 times higher in males. Furthermore, among male blood donors with an identified risk factor, the proportion of MSM increased from 16.7% in 2007 to 64.9% in 2022 [58].

In Ankara, the capital of Turkey, Aydogan et al. [59] reported that syphilis serologic testing was performed on 588 patients. Among these patients, 61 (10,4%) were positive for both the rapid plasma reagin (RPR) test and the treponema pallidum hemagglutination assay (TPHA), while 67 (11.4%) were RPR-negative but TPHA-positive. The distribution of RPR titers was: 1:2 (n = 2), 1:4 (n = 6), 1:8 (n = 12), 1:16 (n = 10), 1:64 (n = 22), 1:128 (n = 2), 1:256 (n = 6), and 1:640 (n = 1). The prevalence of syphilis coinfection was 31.6% before 2019 and ranged from 19.2% to 27.3% in the years after, summing up the prevalence of 22.3% after 2019. Additionally, syphilis coinfection was significantly more common in males (27.7%) than in females (9.0%) (*p* = 0.001). However, there were no significant differences in mean HIV log viral load (3.4830 versus 3.7859; *p* = 0.487) or mean age (40.9 versus 41.0 years; *p* = 0.970) between patients with and without syphilis coinfection [59].

In summary, syphilis notification and seropositivity rates have risen significantly across Europe over the past decade.

### 3.3. Americas

The highest number of syphilis cases were reported in the Americas, according to the WHO Region of the Americas [44].

#### 3.3.1. During COVID-19 Pandemic

##### USA

In New York City, USA, Braunstein et al. [60] reported that the total number of confirmed syphilis cases was 402 in 2019, compared to 324 in 2020, representing a 19% reduction [60].

Similarly, Pagao et al. [61] reported a 0.9% decrease in the incidence of primary and secondary syphilis in the United States during the period from 4 March to 20 May 2020 compared with the corresponding period in 2019 [61].

In contrast, Chang et al. [62] observed a 19% increase in the number of patients diagnosed with syphilis during the COVID-19 pandemic compared with the pre-pandemic period in Southern California, USA [62].

Overall, studies from the United States demonstrated heterogeneous trends during the COVID-19 pandemic, with some regions reporting temporary declines in notified cases, whereas others observed an increase in syphilis diagnoses, likely reflecting regional differences in healthcare access, testing practices, and sexual behaviour.

#### 3.3.2. After the COVID-19 Pandemic

##### USA

Htet et al. [63] revealed in their study that up to January 2021, among 4748 articles identified, 23 studies reported a syphilis prevalence ranging from approximately 0% to 18% in older adults [63]. In King County, Washington, Berzaklns et al. [64] observed in their analysis of data from 2007 to 2022 that among cisgender women, overall syphilis incidence remained stable from 2007 to 2010, but then increased by about 16.3% per year from 2010 to 2020, and surged by 90.1% per year from 2020 to 2022. For cisgender women, overall syphilis incidence remained stable. Early-stage syphilis rose gradually from 2007 to 2017 (≈ 18.0%/year) and then increased rapidly from 2017 to 2022 (≈ 62.5%/year). By contrast, the rise in late/unknown-duration syphilis among cisgender women was delayed. After an early nonsignificant decline or stability, incidence increased more slowly and only began to grow markedly later in the period. Among cisgender MSM, all-stage syphilis incidence increased steadily over the period by about 7.0% per year between 2007 and 2022. In the group of cisgender men who have sex with women, syphilis cases were observed to increase by 25.0% per year (95% CI, 14.0–37.0%) from 2014 to 2022 [64]. At the University of Nebraska Medical Centre, Huang et al. [65] reported that, from 2021 to 2023, 21 patients with ocular syphilis (34 eyes of 21 patients) were included. The median baseline visual acuity (VA) was 6/60 in the methamphetamine-use group, which was significantly worse than in the non-methamphetamine group, where the median VA was 6/18 (*p* = 0.0136). During follow-up, the non-methamphetamine group showed significant improvement in VA: from baseline to month 3rd (6/18 → 6/7.5, *p* = 0.001) and from baseline to month 6th (6/21 → 6/6, *p* = 0.002). In contrast, the methamphetamine group did not show statistically significant improvement: VA changed from 6/120 at baseline to 6/24 at follow-up (*p* = 0.225) [65]. Rankin et al. [66] reported that syphilis cases rose substantially in the United States between 2018 and 2022—a 78.9% increase over those five years, and a 17.0% increase just in the last year alone. Syphilis showed the most dramatic change among common STIs. The highest rates were observed in the Midwest and Southern regions of the United States. Patients who used crack had 62% lower odds of syphilis infection compared to cocaine users (OR = 0.38, *p* < 0.001). Heroin intake was associated with 43% lower odds of syphilis infection (OR = 0.57, *p* < 0.001). In contrast, injection drug use was linked to a significant increase in odds of syphilis infection (OR = 1.89, *p* < 0.001). Meanwhile, methamphetamine intake showed the strongest association: users had over three times higher odds of syphilis infection compared to cocaine users (OR = 3.34, *p* < 0.001) [66]. The risk factors associated with the widespread increase in syphilis included lack of health insurance, low socioeconomic status, high-risk sexual behaviour, domestic violence, unstable housing, substance use, and being non–English–speaking [67,68].

Regarding the recent data from the USA show that cases of primary and secondary syphilis declined by 22% in 2024 [69].

To summarize, the data show that syphilis remains a significant public health concern in the USA, with rising incidence across several populations and strong associations with substance use and socioeconomic and structural vulnerabilities.

### 3.4. Asia

#### 3.4.1. During the COVID-19 Pandemic

In China, Wu et al. [70] reported a substantial decline in syphilis prevalence in both 2020 and 2021, with reductions of 13.32% and 10.41%, respectively, compared with the pre-COVID-19 pandemic year (2019). Furthermore, the number of registered syphilis cases decreased by 17.95% in 2020 and by 20.41% in 2021 relative to 2019 [70].

In Japan, Komori et al. [71] reported that the number of patients diagnosed with syphilis decreased by 55.4% in 2020 (3046 cases) compared with the average of 2018–2019 (7007 cases in 2018 and 6642 cases in 2019), and by 30.6% in 2021 (4735 cases) relative to the 2018–2019 average [71].

In Taiwan, Chia et al. [72] reported that the annual number of newly diagnosed syphilis cases declined during the COVID-19 pandemic, from 6591 cases in 2018 and 6294 cases in 2019 to 5776 cases in 2020, representing an 8% reduction in 2020 compared with 2019 [72].

In contrast, Sambyal et al. [73] reported once again an increasing trend in syphilis cases in the Jammu and Kashmir region of India following the COVID-19 lockdown (8 cases in 2020), with 30 cases reported in 2021 and 42 in 2022, compared with 54 cases reported before the pandemic in 2019 [73].

Overall, studies from Asia predominantly reported a decline in syphilis notifications during the COVID-19 pandemic, although regional differences were observed, with some areas demonstrating a subsequent increase following the relaxation of public health restrictions.

#### 3.4.2. After the COVID-19 Pandemic

In mainland China from 2007 to 2017, Huang et al. [74] reported a significant upward trend in the incidence of syphilis, rising from 15.8834 per 100,000 to 34.4867 per 100,000. Over this period, the total number of new HIV infections (205,084) and syphilis cases (921,279) were highest in the 20–30-year-old age group, with incidence decreasing as age increased beyond 20 years. There was a positive correlation between HIV infection and syphilis incidence (*p* < 0.05). In 2017, the observed incidence rates of syphilis cases in mainland China showed a substantial increase of 127.92% compared to previous years [74].

In Henan Province, China, Zhang et al. [75] observed that, from January 2016 to December 2022, 7030 pregnant women were infected with syphilis. The risk factors for adverse pregnancy outcomes were the occurrence of abnormalities during pregnancy (OR = 5.001, 95% CI: 4.203–5.951), a history of syphilis infection (OR = 1.207, 95% CI: 1.035–1.409), not receiving standard treatment for syphilis (OR = 1.370, 95% CI: 1.095–1.716), a titer ≥1:8 at diagnosis (OR = 1.350, 95% CI: 1.079–1.690), and a titer ≥1:8 before delivery (OR = 1.985, 95% CI: 1.463–2.694) [75].

In Busan, Korea, Lee et al. [76] observed that the incidence of syphilis cases per 100 person-years (PY) gradually decreased from 2.43 (95% CI: 0.79–7.42) in 2005–2007 to 1.85 (95% CI: 1.08–3.17) in 2014–2016. In contrast, it increased to 3.0 (95% CI: 1.99–4.53) in 2017–2019 and further to 3.33 (95% CI: 2.26–4.89) in 2020–2022. The authors identified the following risk factors for incidence syphilis: a positive treponemal test at baseline (HR = 2.33, 95% CI: 1.48–3.67, *p* < 0.001), men who have sex with men (HR = 2.36, 95% CI: 1.34–4.16, *p* = 0.003), young age (≤30 years versus >50 years; adjusted HR = 6.27, 95% CI: 2.38–16.56, *p* < 0.001), and a history of incarceration (HR = 2.62, 95% CI: 1.21–5.67, *p* = 0.015) [76].

In India, Golia et al. [77] conducted a meta-analysis that included 41 studies involving 1,860,594 blood donors. The pooled prevalence rates for HIV, HBV, HCV, syphilis, and malaria among Indian blood donors were 0.12%, 0.91%, 0.28%, 0.14%, and 0.01%, respectively. The highest prevalence of syphilis cases was noted in the northern part of the country, with rates ranging from 0.05% to 0.48% [77].

In Swat, Pakistan, Shah et al. [78] reported that, among 94,716 healthy blood donors, a total of 2292 (2.42%) transfusion-transmissible infections (TTIs) were detected. Specifically, these included 873 (0.92%) cases of HCV, 637 (0.67%) cases of HBV, 220 (0.23%) cases of HIV, and 562 (0.59%) cases of syphilis. Both syphilis and HIV cases showed a significant increase in 2024 (*p* < 0.001). The prevalence of syphilis cases increased by 1.12% in the 34–49-year age group [78].

In summary, after the COVID-19 pandemic, the outcomes showed substantial geographic and temporal differentiation in syphilis epidemiology across Asia, with increasing incidence reported in China and Korea and notable prevalence among blood donors in India and Pakistan, particularly among younger and other identified high-risk populations.

### 3.5. Brazil

In Brazil, studies consistently demonstrate that gestational syphilis remains a major public health concern. Moura et al. [79] reported 229,558 cases of gestational syphilis between 2007 and 2017, including 7400 cases in the state of Ceará, and showed that implementation of the *Rede Cegonha* strategy was associated with significant differences according to maternal education, clinical classification, and gestational age [79]. Although prenatal syphilis screening improved substantially in Brazil between 2013 and 2019, treatment coverage among infected pregnant women declined during the same period, indicating persistent gaps in antenatal care and syphilis management [80].

Studies conducted in vulnerable populations also reported a high burden of syphilis. Among homeless individuals in a major Central–Western Brazilian city, the prevalence reached 22% by rapid testing, with younger age, previous syphilis testing, genital lesions, and recent crack use identified as independent risk factors [81]. Barbosa et al. [82] found a prevalence of *Treponema pallidum* infection of 6.04% among Brazilian women, with infection being associated with multiple sexual partners, lower income, lower educational level, previous sexually transmitted infections, and inconsistent condom use [82]. Similarly, Camargo Junior et al. [83] reported a syphilis prevalence of 14.4% among sex workers (group of 104 sex workers) in the state of Goiás, highlighting the high burden of infection in this vulnerable population [83].

### 3.6. Argentina

Barris et al. [84] investigated the prevalence of sexually transmitted infections among women undergoing voluntary termination of pregnancy and found that 23.4% of the analysed patients were diagnosed with an STI. Syphilis accounted for six cases, including four primary infections and two reinfections, highlighting the importance of routine STI screening in this population [84].

### 3.7. Africa

In South Africa, Kufa et al. [85] demonstrated that the strong association between HIV and syphilis prevalence observed in 2022 is consistent with findings from sub-districts (townships/areas) that experienced the largest increases in syphilis prevalence. At the district level, antenatal HIV prevalence in 2022 was positively correlated with syphilis prevalence (Spearman’s Rho = 0.42; *p* = 0.0018). Notably, the six districts with the highest HIV prevalence in 2010 showed a marked increase in syphilis prevalence from a median of 0.1% (IQR 0.0–0.4%) in 2010 to a median of 5.2% (IQR 4.2–5.8%) in 2022 [85].

In Ethiopia, Tura et al. [86] reported a syphilis prevalence of 6.2% among 6085 female sex workers. Factors significantly associated with syphilis infection included the following: age 30–34 years (AOR = 2.64; 95% CI: 1.40–4.98), 35–59 years (AOR = 4.70; 95% CI: 2.50–8.86), divorced or widowed women (AOR = 1.37; 95% CI: 1.03–1.82), no formal education (AOR = 3.38; 95% CI: 2.34–5.11), with only primary first cycle education (grades 1–4) (AOR = 2.77; 95% CI: 1.79–4.30), with primary second cycle education (grades 5–8) (AOR = 1.80; 95% CI: 1.21–2.69) [86]. In Northeast Ethiopia, Metaferia et al. [87] reported that, among 360 female sex workers, 45 participants (12.5%) were diagnosed with syphilis. Of these, 16/360 (4.4%) were co-infected with HIV and syphilis, and 6/360 (1.7%) had triple infection with HIV, HBV, and syphilis. Furthermore, the authors reported that age, having children, marital status, sharing of sharp materials, history of condom breakage, and genital discharge were all positively and significantly associated with *T. pallidum* infection (*p* < 0.05) [87]. In Ethiopia, Wariso et al. [88] reported that, among 6085 female sex workers surveyed between 2019 and 2020, the prevalence of infection was 18.2% for HIV, 6.2% for syphilis, 2.5% for HBV, and 0.5% for HCV. The odds of having at least one STI were significantly lower among educated female sex workers, whereas a history of condom breakage was associated with an increased risk of STI acquisition [88]. In Ethiopia, Alemu et al. [89] reported that, in their 2018 study of 381 female sex workers, the majority of participants were aged 20–24 years, single, and resided in urban areas. The seroprevalence of syphilis among these female sex workers was 4.2% [89].

Okiria et al. [90] showed that 1 in 5 (19.8%) of 409 female sex workers and sexually exploited adolescents experienced a condom breakage during vaginal or anal sex in the last six months, in Nimule town at the border of South Sudan and Uganda. HIV prevalence was 24.0% (95% CI: 19.4–28.5), and that of syphilis was 9.2% (95% CI: 6.5–11.9). In multivariable analysis, there was a strong association between HIV infection and active syphilis (adjusted odds ratio: 6.99; 95% CI: 2.23–21.89) [90].

In Mthatha, South Africa, Tshaka et al. [91] found that the prevalence of syphilis, 2018–2019, as measured by RPR positivity, was highest in the 30–39 age group (12.12%), with no positive cases recorded in the <20, 50–59, and ≥60 age groups. Overall, syphilis presented a relatively low burden across all age groups, which, as the authors concluded, may reflect either effective screening and treatment programmes or underdiagnosis in certain populations [91].

In Kenya, Nzivo et al. [92] reported an overall syphilis prevalence of 2.24% (*p* = 0.0001) among female sex workers. Syphilis was detected in 2 of 44 (4.5%) HIV-positive participants and in 4 of 224 (1.8%) HIV-negative participants. Syphilis infection was observed only in the 25–29 and ≥35-year age groups, which also had the highest HIV prevalence. HIV/syphilis co-infection occurred exclusively in the 25–29-year age group. Human herpes virus 8 (HHV-8)/syphilis co-infection was equally distributed between the 25–29-year (50%) and ≥35-year (50%) age groups. Overall, HHV-8/syphilis co-infection was rare (3%). Although consistent condom use is an effective method for preventing HIV and syphilis, it was not associated with HHV-8 transmission in this study, suggesting that condom promotion would have minimal impact on HHV-8 control. Syphilis was not significantly associated with HHV-8 infection (OR 1.52, 95% CI 0.27–8.47) [92].

Across seven regions of Tanzania, Vu et al. [93] reported an overall syphilis prevalence of 8% in 2013, with a significantly higher burden observed in the following cities: Iringa (11%), Mbeya (13%), and Shinyanga (12%) [93].

Hakim et al. [94] observed that 7.3% of 838 female sex workers had active syphilis in the previous 12 months. The study enrolled 838 female sex workers in South Sudan between November 2015 and March 2016. The authors concluded that the high prevalence of HIV and active syphilis among female sex workers in Juba highlights the urgent need for targeted HIV and STI services for this population [94].

Overall, the outcomes showed a significant and heterogeneous burden of syphilis across sub-Saharan Africa, especially among female sex workers, with prevalence varying considerably between countries and settings and frequent co-occurrence with HIV.

### 3.8. Australia, New Zealand

According to the National Notifiable Diseases Surveillance System and the Australian Bureau of Statistics [95], a total of 5866 diagnoses of infectious syphilis were registered across Australia in 2024. This represents approximately twice the number of syphilis diagnoses recorded a decade earlier [95]. In Victoria, Australia, Borg et al. [96] reported that infectious syphilis notifications were approximately five times higher in 2020 than in 2010 (1440 in 2020 compared with 289 cases in 2010). Interestingly, a more than sevenfold increase was observed among women (25 cases in 2010 compared with 186 in 2020) [96]. In South Australia, Sisnowski et al. [97] observed a high prevalence of both injection drug use (IDU) and non-injection drug use (non-IDU) among patients with syphilis diagnosed outside specialist STI outpatient clinics, in the period of 1 July 2022–30 September 2023. Females and heterosexual individuals were more frequently reported as drug users than MSM. The researchers identified 450 probable and confirmed cases of infectious syphilis over the study period. Of these, 14% (n = 62/450) were women, and 57% were diagnosed outside STI outpatient clinics. Their study revealed a notable prevalence of drug use among syphilis cases: 17% of patients reported non-IDU and 10% reported IDU [97]. According to the New Zealand Institute for Public Health and Forensic Science (PHF Science) Ltd. [98], the number of registered infectious syphilis cases in New Zealand remained elevated in 2024, with 774 cases recorded—an increase from 739 cases in 2023 [98].

In total, the results demonstrated that infectious syphilis remains an important and growing public health concern. In Australia, approximately twice as many diagnoses were reported in 2024 as a decade earlier, and substantial increases were observed among women and people who used illicit drugs.

## 4. Diagnostic Procedures

The diagnostic process for syphilis includes direct detection methods such as dark-ground microscopy (DGM), polymerase chain reaction (PCR), immunohistochemistry, and silver staining, which are particularly useful for diagnosing early syphilis (primary and secondary stages). DGM and PCR typically use exudate or discharge from primary chancres or secondary skin/mucosal lesions. IHC and silver staining are applied to tissue biopsies (e.g., from skin lesions). PCR can also be used on placental tissue in cases of suspected congenital syphilis. However, these direct detection methods generally lack sufficient specificity and sensitivity when applied to blood samples or cerebrospinal fluid. Moreover, they cannot be used to monitor treatment response, as they detect the presence of the organism (or its DNA) rather than host antibody levels, which are followed serologically for assessing treatment efficacy [99,100].

### 4.1. Nontreponemal and Treponemal Tests

For proper diagnosis of syphilis, it is mandatory to evaluate at least two blood serological tests, like one nontreponemal test and one treponemal test [101]. Non-treponemal tests utilise a complex antigen consisting of cardiolipin, lecithin, and cholesterol. Examples include the VDRL test, the RPR test, and the Toluidine Red Unheated Serum Test (TRUST) [102]. On the other hand, treponemal tests use recombinant antigens of treponemal, which detect IgM and IgG. Examples of treponemal tests are *T. pallidum* Particle Agglutination test (TPPA), *T. pallidum* Haemagglutination test (TPHA), Fluorescent Treponemal Antibody Absorption test (FTA-abs test), Treponemal Enzyme Immunoassay (EIA), Enzyme-Linked Immunosorbent Assay (ELISA), IgG or IgM immunoblot test for *T. pallidum*, or Chemiluminescence Immunoassay (CLIA) [102,103].

Treponemal tests typically become positive during the 3rd to 4th week of infection. IgM antitreponemal antibodies appear earlier, around the 2nd week of infection [104,105]. However, it has been proven that IgM antibodies can be detected in around 60% of patients with primary syphilis [106,107]. The levels of IgM antibodies usually decrease after 4 months of treatment, but may remain elevated for longer if the disease is left untreated [108]. Disease activity is not correlated with treponemal test titers, in contrast to nontreponemal test titers. In the vast majority of patients with syphilis, treponemal titers remain positive for life. To confirm a positive treponemal test result, a nontreponemal test is necessary [109].

Serological scar refers to a persistent, low-level positive treponemal antibody titer (e.g., TPHA, TPPA) that remains detectable following successful treatment for syphilis [110].

On the other hand, a serofast state, which is defined as a condition in which a patient’s non-treponemal antibody titers (RPR or VDRL) remain persistently low or without a fourfold decline after adequate treatment, despite the complete resolution of clinical symptoms [111,112].

### 4.2. Conventional and Reverse Algorithms

In the conventional algorithm, a nontreponemal test (like RPR or VDRL) is used as the initial screening tool. Subsequently, this is automatically followed by a treponemal test to confirm the diagnosis of syphilis [113].

In the reverse algorithm, this pathway initiates with a treponemal immunoassay (like TPHA or TPPA) as the initial screening. If the screening test is reactive, a quantitative nontreponemal test (for instance VDRL or RPR) is carried out. If the results are incompatible (e.g., initial treponemal screen is positive, but the RPR or VDRL is negative), a second, different treponemal test (than used in the beginning) is typically used to resolve the status [113].

### 4.3. Differential Diagnosis

Syphilis is known as “the great imitator” due to its highly variable clinical presentations, particularly skin lesions, which arise from inflammatory responses to the replication of spirochetes [114]. The skin manifestations of syphilis are characterised by their remarkable diversity. In the course of this venereal disease, skin lesions may present as macular, maculopapular, papular, papulosquamous, pustular, nodular, or lichenoid [115]. The maculopapular eruption in syphilis can resemble that of lichen planus [116] or psoriasis [116,117,118]. Unlike lichen planus, however, syphilitic skin lesions are usually nonpruritic and characteristically affect the palms and soles [116]. In addition, the vesiculonecrotic and papulonecrotic lesions seen in syphilis can resemble those of pityriasis lichenoides et varioliformis acuta (PLEVA) [119]. Widespread papules of varying sizes with central necrosis, along with plaques on the face, trunk, and extremities, may mimic pityriasis lichenoides chronica (PLC) [120,121]. Syphilitic target-like erythematous plaques and papules may mimic the skin lesions seen in erythema multiforme [122]. Anetoderma-like skin lesions in secondary syphilis may present as nodules, herniated papules, and erythematous plaques on the cervical area, trunk, and arms [123].

Patients infected with the monkeypox virus may develop skin lesions similar to those of primary syphilis, such as a rounded ulcer with an infiltrated border [124,125,126].

## 5. Conclusions

Syphilis remains a major and re-emerging public health challenge worldwide, with a steadily increasing incidence observed in many regions. Owing to its diverse clinical manifestations, the disease continues to pose considerable diagnostic challenges and should always be considered in the differential diagnosis of a wide range of dermatological and systemic disorders. Known as the “great imitator of skin lesions”, syphilis can mimic numerous common dermatological conditions, including psoriasis, pityriasis rosea, mycosis fungoides, and others, which frequently leads to misdiagnosis. Early recognition and accurate diagnosis are essential to prevent disease progression, ongoing transmission, and congenital infection.

Despite substantial advances in diagnostic methods and surveillance systems, significant gaps remain in epidemiological reporting, access to testing, and timely treatment, particularly among vulnerable populations. Strengthening surveillance programmes, improving access to early diagnosis, and expanding public health initiatives focused on STI prevention, education, and awareness are essential to limit the continuing spread of syphilis and reduce its global health burden.

## Data Availability

No new data were created or analysed in this study. Data sharing is not applicable to this article.

## Abbreviations

age-standardised incidence (ASIR), adjusted odds ratio (AOR), sexually transmitted infections (STIs), congenital syphilis (CS), European Union and the European Economic Area (EU/EEA) countries, Benzathine benzylpenicillin G (BPG), central nervous system (CNS), intramuscularly (i.m.), intravenously (i.v.), million units (MU), World Health Organisation (WHO), people living with HIV (PLWH), men who have sex with men (MSM), European Centre for Disease Prevention and Control (ECDC), blood donors (BDs), rapid plasma reagin (RPR), treponema pallidum hemagglutination assay (TPHA), Venereal Disease Research Laboratory (VDRL), relative risk (RR), visual acuity (VA), transfusion-transmissible infections (TTIs), injection drug use (IDU), non-injection drug use (non-IDU), dark-ground microscopy (DGM), polymerase chain reaction (PCR), *T. pallidum* Particle Agglutination test (TPPA), *T. pallidum* Haemagglutination test (TPHA), Fluorescent Treponemal Antibody absorption test (FTA-abs test), Treponemal Enzyme Immunoassay (EIA), Enzyme-Linked Immunosorbent Assay (ELISA), Chemiluminescence Immunoassay (CLIA), pityriasis lichenoides et varioliformis acuta (PLEVA), pityriasis lichenoides chronica (PLC), computed tomography (CT), immunohistochemistry (IHC), Human herpes virus 8 (HHV-8).
